# Chemoradiation-Altered Micromilieu of Glioblastoma Cells Particularly Impacts M1-like Macrophage Activation

**DOI:** 10.3390/ijms26146574

**Published:** 2025-07-08

**Authors:** Mona Shojaei, Benjamin Frey, Florian Putz, Rainer Fietkau, Udo S. Gaipl, Anja Derer

**Affiliations:** 1 Translational Radiobiology, Department of Radiation Oncology, Universitätsklinikum Erlangen, Friedrich-Alexander-Universität Erlangen-Nürnberg (FAU), 91054 Erlangen, Germany; mona.shojaei@uk-erlangen.de (M.S.); benjamin.frey@uk-erlangen.de (B.F.); udo.gaipl@uk-erlangen.de (U.S.G.); 2 Department of Radiation Oncology, Universitätsklinikum Erlangen, Friedrich-Alexander-Universität Erlangen-Nürnberg (FAU), 91054 Erlangen, Germany; florian.putz@uk-erlangen.de (F.P.); rainer.fietkau@uk-erlangen.de (R.F.); 3 Comprehensive Cancer Center Erlangen-EMN, 91054 Erlangen, Germany

**Keywords:** glioblastoma, chemoradiation, macrophages, TAM, immune checkpoint molecule, PD-1, PD-L1/L2

## Abstract

Glioblastoma is a highly aggressive brain tumor with an overall poor prognosis due to its immunosuppressive tumor microenvironment (TME). Microglia and tumor-associated macrophages (TAMs) with pro-tumorigenic properties are dominant populations of immune cells in the glioblastoma TME. To date, several studies targeting TAMs to fight tumor progression in different tumor entities have been initiated. However, the impact of standard therapy schemes of glioblastoma cells on macrophage polarization, activation, and phagocytosis remains controversial. The same applies to the relevance of PD-1/PD-L1 blockade in the interaction between macrophages and tumor cells. Our study, therefore, investigated patient-oriented treatment of GLIOBLASTOMA by examining the phagocytic capacity of polarized M1- and M2-like macrophages using GL261-luc2 tumor cells as a preclinical model system. Additionally, we analyzed the expression of activation and immune checkpoint markers on these macrophage subtypes following contact with tumor cells and their microenvironment. These factors were also determined after PD-1 blockade was initiated. The analyses revealed that the immunoregulatory M2-like macrophages generally exhibited a higher phagocytosis rate than the pro-inflammatory M1-like macrophages; however, this was not influenced by the pretreatment of glioblastoma cells with chemo- or radiotherapy. This could not be improved by blocking the PD-1 receptor. Furthermore, there were no modulations in the expression of differentiation, activation, or immune checkpoint molecules of M1- and M2-like macrophages after cell-to-cell contact with glioblastoma cells. But the medium conditioned by tumor cells strongly altered M1-like macrophages toward a more activated state, whereas M2-like cells were only mildly influenced. This was further enhanced by tumor cell treatment, with the most prominent effect after irradiation. These results suggest that conventional GLIOBLASTOMA tumor cell treatment affects the immunogenic status of macrophage subtypes, which is relevant for enhancing the anti-tumor immune response in brain tumors.

## 1. Introduction

Glioblastoma is a highly malignant grade IV astrocytoma with an overall survival rate of only about 15 months and a particularly high risk of relapse, despite long-term research and innovative treatment strategies. To date, conventional fractionated radiotherapy combined with chemotherapy (temozolomide) is still the standard of care, as it has been for decades. This is despite years of clinical studies and investigations. The therapy is complicated by factors such as the tumor localization, its heterogeneity, the overall low mutational burden, and its immunosuppressive character [1]. Immunotherapy is one promising tool in fighting cancer that has been successful in many tumor entities in subgroups of patients. One key option is the targeting of immune checkpoints. Immune checkpoints naturally function as regulatory feedback mechanisms following immune activation to prevent immune overstimulation and the uncontrolled proliferation of reactive lymphocytes, thereby maintaining physiological homeostasis after infection or inflammation [2]. Tumors exploit these mechanisms to limit the function of anti-tumor effector cells and evade immune surveillance. Cytotoxic T Lymphocyte Antigen 4 (CTLA-4) and Programmed Cell Death Protein 1 (PD-1)/Programmed Death Ligand 1 (PD-L1) are two prominent immune checkpoints known to impact the outcome of several tumor entities. The ligand PD-L1 can be expressed by activated T and B cells, but also by glioblastoma cells themselves [3], as well as microglia and TAMs. Additionally, standard radiotherapy (RT) has been shown to have unwanted side effects on tumor cells concerning the expression of the immune checkpoint molecules programmed death ligand 1 (PD-L1) and PD-L2 [4]. Its receptor, PD-1, is mainly found on activated T cells. The interaction between this receptor–ligand pair inhibits the proliferation and cytolytic function of effector T cells, which subsequently has an immunosuppressive effect on a potential T-cell response and thus is beneficial to the tumor. Therefore, one immunotherapeutic approach has been to target the PD-1/PD-L1 axis with inhibitory antibodies to block counter-regulatory signaling in T cells and promote anti-tumor immune responses, which has been highly successful for a subset of patients in various tumor entities, such as lung cancer. However, no breakthrough has yet been achieved for glioblastoma.

Under healthy conditions, the major immune sentinels of the central nervous system (CNS) inside the brain are microglia and astrocytes [5]. As such, they manage immune surveillance, contribute to maintaining the blood–brain barrier (BBB), and obtain homeostasis. Yet, pathological conditions foster the escape of immune surveillance. Glioblastomas can evade the immune system through several suppressive mechanisms, such as the expression and/or secretion of immune checkpoint molecules, cytokines, or chemokines, and the subsequent recruitment and activation of suppressive immune cells such as regulatory T cells (Treg), myeloid-derived suppressor cells (MDSCs) or tumor-associated macrophages (TAMs). TAMs comprise about half of the glioblastoma microenvironment [6,7], including infiltrating macrophages and resident microglia. The recruitment and polarization of these immune cells are especially influenced by the tumor microenvironment (TME). A study by Kvisten et al. investigated the localization of TAMs within glioblastoma tissue and found them primarily in the central tumor area rather than in the infiltration zone. In addition, they confirmed that TAMs in glioblastoma differ in morphology, which corresponds to different activation states [8]. Furthermore, the population of TAMs seems to vary between slow- and fast-growing glioblastoma. These findings give evidence of a role for TAMs in the therapy resistance of glioblastoma. In general, the mechanisms of action of TAMs associated with tumor progression in other cancer entities include modulation of the immune response, modification of the TME, and direct support of tumor cells. Thus, several therapeutic approaches target TAMs, ranging from TAM depletion [9] to blocking specific TAM functions in immunosuppression or angiogenesis [9], enhancing phagocytosis [10], or reprogramming TAM polarization [11]. The polarization of macrophages into M1- and M2-type is essential for their immunological function. M1 macrophages are considered pro-inflammatory and anti-tumorigenic, while M2 macrophages, designated as TAMs, may contribute to tumor progression through several regulatory mechanisms as described above [11]. TAMs promote tumor growth by suppressing anti-tumor immunity through immune checkpoint molecules, such as the PD-1/PD-L1 axis. Using a CT26 colon carcinoma model, Gordon et al. discovered that TAMs expressing PD-1 correlate negatively with the phagocytic potency of tumor cells. They also found that blocking PD-1/PD-L1 increases macrophage phagocytosis, reduces tumor growth, and enhances survival [12].

Significantly, TAM polarization is influenced not only by the tumor itself but also by treatment-induced changes in the TME. In glioblastoma, studies investigating the effects of TMZ and radiotherapy (RT) on macrophage polarization have produced contradictory results. The effect of TMZ appears to vary depending on the context. TMZ has been shown to both induce M2 polarization [13,14] and promote M1 activation via HMGB1 release through secretory autophagy [15]. Other reports suggest that TMZ sustains CXCL2/CXCR2 signaling, contributing to angiogenesis, tumor progression, and recruitment of M2-like macrophages [16]. Additionally, glioblastoma resistance to TMZ may be attributed to M2-type hypoxia-associated macrophages [16]. Similarly, RT has been linked to both immunostimulatory and immunosuppressive effects. In a head and neck squamous cell carcinoma (HNSCC) model, RT increased the polarization of both M1 and M2, but shifted towards an immunosuppressive phenotype [17]. Irradiation of lung tumor cells resulted in the secretion of exosomes that promoted M2 polarization [18]. A systematic review analyzed 29 summary articles regarding the role of M1 and M2 macrophages in response to the radiation dose. It concluded that high-dose irradiation (>10 Gy) favors angiogenesis and tumor growth through early M2-polarized TAMs, whereas low to moderate dose irradiation (1–10 Gy) may stimulate phagocytosis [19] and promote an M1-associated phenotype [20]. Consistently, recent work has shown that radiation induces M2 polarization via STAT5-dependent glutamine synthetase expression, using a single dose of 10 Gy [21]. In murine glioma models, Akkari et al. demonstrated radiation-specific and stage-dependent gene expression patterns in monocyte-derived macrophages [22]. These and other studies demonstrate a significant impact of glioblastoma treatment on TAM polarization. Taken together, a growing body of literature supports a key role for M2 macrophages in chemo- and radioresistance in glioblastoma [23]. However, the results are still conflicting, suggesting a high degree of context dependency, and the impact of combining both CT and RT in chemoradiation remains poorly understood.

The high abundance of TAMs in the immunosuppressive tissue of glioblastoma, combined with the inhibitory function of immune checkpoint signaling, led us to investigate how the dual-modality standard treatment of RT and TMZ affects macrophage polarization and activation. Specifically, we aimed to analyze the regulatory function of the PD-1/PD-L1 interaction in polarized M1- and M2-like macrophages, with a focus on the phagocytic capacity of glioblastoma cells. Additionally, we aimed to elucidate the changes in the expression of activation and immune checkpoint markers in M1- and M2-like macrophages following treatment with glioblastoma. By dissecting these interactions, we aim to gain a deeper understanding of how standard chemoradiation of glioblastoma cells shapes M1-like and M2-like macrophage activation and immune checkpoint expression and identify potential points of intervention for future immune-targeted therapies in glioblastoma.

## 2. Results

### 2.1. Neither Chemoradiation of Glioblastoma Cells nor PD-1 Blockade Affects the Differentiation Status or Immune Checkpoint Expression of M1- and M2-like Cells

Various preclinical studies have demonstrated the role of PD-1 on myeloid cells in tumor treatment [12,24,25], providing evidence for studies on PD-1 receptor/ligand blockade on TAMs in glioblastoma. To investigate the impact of chemoradiation on glioblastoma cells, as well as the effect of PD-1/PD-L1 blockade on TAM differentiation and activation, we prepared syngeneic co-culture assays using GL261-luc2 glioblastoma cells with M1-like and M2-like macrophages. GL261 cells are IDH1-wildtype cells and can thus model primary (IDH-wt) glioblastoma [26]. M1- (classically activated) and M2-like (alternatively activated) macrophages were differentiated from the bone marrow of C57BL/6 mice via cytokine stimulation. Their immunogenic status was then determined using flow cytometry by examining various surface markers to detect the differentiation, activation, and expression of immune checkpoint molecules. The tumor cells were treated with temozolomide (TMZ) as chemotherapy (CT), fractionated radiotherapy (RT), or a combination of the two (RCT) in a patient-oriented manner. Mock-treated cells served as controls.

**Figure 1:**
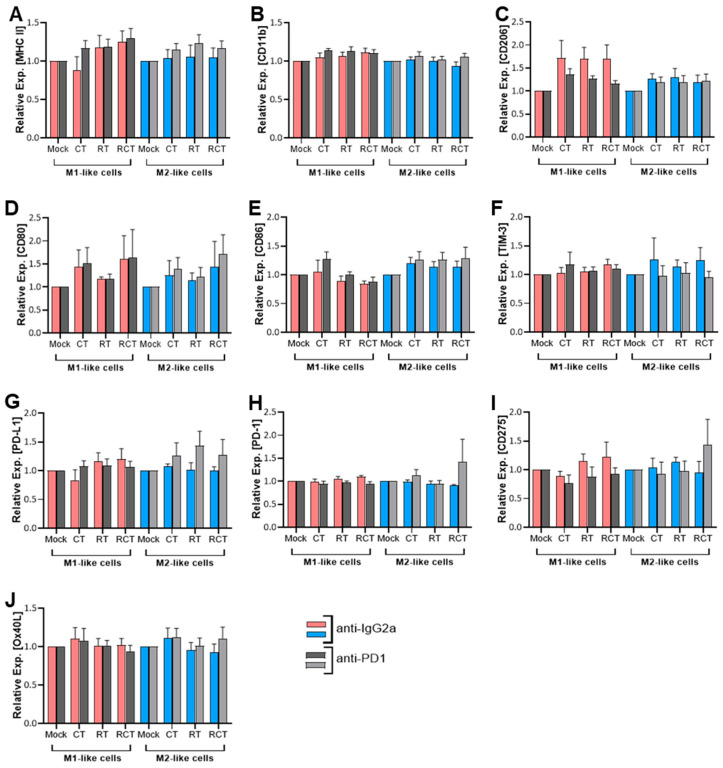
Surface expression of activation and immune checkpoint molecules on M1-like (red and dark grey columns) and M2-like (blue and light grey columns) macrophages after co-culture with treated glioblastoma cells. GL261-luc2 glioblastoma cells were treated with temozolomide (CT), irradiation (RT), the combination (RCT), or left untreated (mock). Bone marrow-derived murine myeloid cells were differentiated and polarized towards M1-like and M2-like macrophages and then co-cultured with treated tumor cells. The surface expression of (**A**) MHCII, (**B**) CD11b, (**C**) CD206, (**D**) CD80, (**E**) CD86, (**F**) TIM-3, (**G**) PD-L1, (**H**) PD-1, (**I**) CD275, and (**J**) OX40L were analyzed via flow cytometry. In addition, anti-PD1 (light and dark grey columns) and anti-IgG2a, serving as the isotype control (red and blue columns), were added. Normalized data are shown for better comparability. Data from five independent experiments are shown as Mean ± SEM. Statistical analysis was performed using a 2-way ANOVA with the Tukey correction for multiple comparisons.

**Figure 2:**
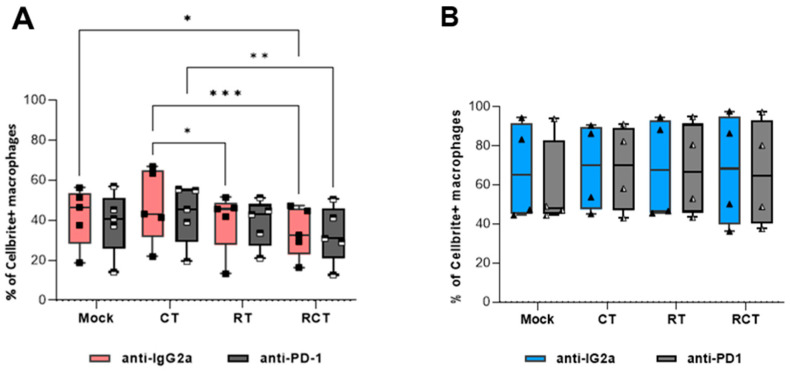
Phagocytosis of GL261-luc2 glioblastoma cells by M1-like (**A**) and M2-like (**B**) macrophages. Tumor cells were treated with temozolomide (CT), irradiation (RT), the combination (RCT), or left untreated (mock). Then, tumor cells were harvested and stained with CellBrite. Bone marrow-derived murine myeloid cells were differentiated and polarized towards either M1-like (**A**) or M2-like (**B**) macrophages. Phagocytosis assays were prepared. To determine the relevance of the PD-1/PD-L1 interaction, anti-PD-1 (light and dark grey columns) or anti-IgG2a as an isotype control (red and blue columns) were added. The percentage of CellBrite-positive macrophages determined the phagocytosis rate. Data are presented as Min to Max; Points represent independent experiments, for (**A**) *n* = 5 and (**B**) *n* = 4. Statistical analysis was performed using a 2-way ANOVA with the Bonferroni correction for multiple comparisons. Asterisks indicate significant differences as follows: ns, *p* = 0.12; * *p* < 0.033; ** *p* < 0.002; *** *p* < 0.001.

Major Histocompatibility Complex II (MHC II) molecules, essential for antigen presentation and thus induction of adaptive immune responses, are induced by factors such as lipopolysaccharide (LPS) and interferon-gamma (IFN-γ) and found to be more highly expressed on M1-like than M2-like cells [27]. Their differential expression results in a more efficient antigen presentation in M1-like macrophages, whereas the lower MHC II level in M2-like cells plays a role in resolving inflammation and fostering tissue repair [28]. Cellular contact of M1-like cells with treated glioblastoma cells resulted in a slight, though not statistically significant, up-regulation of MHC II after treatment with the most prominent effect occurring post-chemoradiation (RCT). This effect was reduced in M2-like cells (Figure 1A). CD11b is an integrin expressed on both M1- and M2-like cells. However, it is activated differently in each cell type, resulting in distinct downstream effects. Nevertheless, no impact on its surface expression could be detected in dependence of tumor cell treatment (Figure 2B). To evaluate the effects of cell–cell contact between M1- or M2-like cells and tumor cells on the differentiation status of macrophages, we chose to determine CD206 as a marker to identify M2 macrophages. Although we found it to be expressed on both subtypes, it was much more prevalent on M2-like cells. But again, no significant alterations were detected after tumor cell treatment. Interestingly, contact with treated tumor cells enhanced the expression of CD206 on M1-like cells. However, this was not statistically significant (Figure 1C). CD80 and CD86 are costimulatory molecules expressed on both macrophage subtypes, with CD80 being more closely associated with M1 macrophages and pro-inflammatory responses. Both molecules are found on tumor-infiltrating lymphocytes (TILs) and TAMs [29,30]. In the glioblastoma TME, higher expression of CD80 and CD86 was associated with shorter progression-free survival (PFS) [30]. Our experiments reveal the expression of both CD80 and CD86 molecules on both macrophage subtypes (Figure 1D,E). Although no significant differences were detected in CD80 expression depending on treatment, a slight increase in CD80 expression was observed on M1-like cells after cell-to-cell contact with CT- and RCT-treated tumor cells.

Since no modulation of activation or differentiation markers on M1- and M2-like cells was detected upon contact with tumor cells, we further evaluated the expression of immune checkpoint molecules to determine the immunogenic profile of these macrophages after tumor cell contact. The expression of PD-1 and PD-L1 on TAMs was associated with a response to PD-1 blockade in different tumor models [25,31]. High levels of T cell immunoglobulin and mucin-domain containing-3 (TIM-3) on tumor-reactive CD8^+^ T cells also correlate with an overall positive response to anti-PD-1 therapy [31]. In the context of TAMs, TIM-3 has been associated with polarization towards the M2 subtype, for example, in anaplastic thyroid cancer [32] and colorectal cancer [33]. Additionally, a poor prognosis regarding overall survival (OS) in various solid tumor entities was described [34,35]. In our analyses, all the described factors were found to be expressed on M1- and M2-like cells (Figure 1F–H). However, neither of these factors was found to be significantly regulated depending on tumor cell treatment. CD275, also known as inducible costimulator ligand (ICOS-L), is expressed by DCs, macrophages, and tumor cells, and interacts with its receptor ICOS on T cells, thereby playing a crucial role in modulating immune responses within the TME. Depending on the context, both anti-tumor T cell activation and pro-tumor regulatory T cell function are possible [36]. A 2022 study showed a correlation between higher ICOS and increased malignancy in gliomas [37], which is why we also analyzed CD275 expression on macrophage subtypes. OX40L is another immune checkpoint with therapeutic implications due to its dual role in immune modulation [38]. OX40L expression modulates adaptive immune responses depending on the TME in glioblastoma [39]. In the context of tumor cell treatment, no alterations in OX40L and CD275 expression were detected between M1- and M2-like cells. The expression was also independent of the tumor cell treatment (Figure 1I,J). Notably, in all conditions, the addition of anti-PD-1 antibodies did not affect the surface expression of either of the investigated molecules (Figure 1).

### 2.2. M2-like Macrophages Have a Higher Capacity to Phagocytize Tumor Cells than M1-like Macrophages, Regardless of Tumor Cell Treatment

Since the cellular contact of tumor cells with macrophages had no significant impact on their activation, differentiation, or immune checkpoint expression, we sought to analyze the phagocytosis of glioblastoma cells in dependence on treatment and macrophage subtype. Murine bone marrow-derived monocytes were polarized into M1- and M2-like macrophages via stimulation with cytokines. GL261-luc2 glioblastoma cells, which were differentially treated, were used as bait. We further tested the effect of PD-L1 expression on phagocytic capacity by adding an anti-PD-1 therapeutic antibody.

Notably, the phagocytosis rate of polarized M2-like macrophages was higher than that of M1-like macrophages (Figure 2). At the same time, there were no differences in the phagocytic capacity of M2-like cells among the treatment conditions (Figure 2B). M1-like macrophages exhibited a significant decrease in tumor cell uptake following chemoradiation. Single treatments revealed that RT was most likely responsible (Figure 2A). In contrast to findings in a colorectal tumor model [12], we could not detect a PD-1/PD-L1-dependent phagocytosis of glioblastoma cells.

### 2.3. The Altered Micromilieu of Treated Tumor Cells Significantly Impacts M1-like Macrophage Activation and Immune Checkpoint Surface Expression

**Figure 3:**
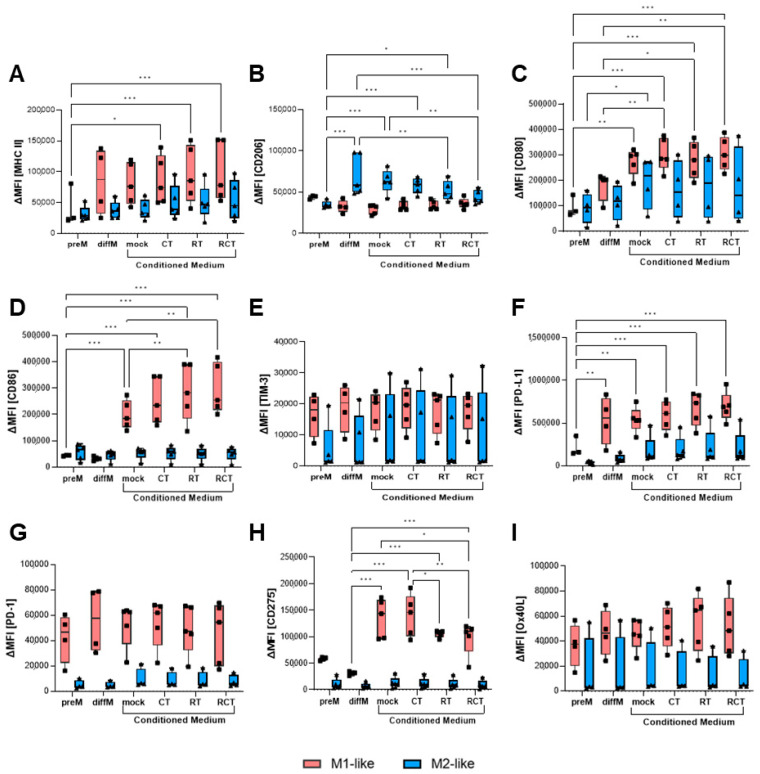
Surface expression of activation and immune checkpoint molecules on M1-like (red boxes) and M2-like (blue boxes) macrophages after incubation with conditioned medium of treated glioblastoma cells. GL261-luc2 cells were treated with temozolomide (CT), irradiation (RT), the combination (RCT), or left untreated (mock), then the supernatant was collected. Bone marrow-derived murine myeloid cells were differentiated and polarized towards M1-like and M2-like macrophages, and incubated with tumor cell medium. The surface expression of (**A**) MHC II, (**B**) CD206, (**C**) CD80, (**D**) CD86, (**E**) TIM-3, (**F**) PD-L1, (**G**) PD-1, (**H**) CD275, and (**I**) OX40L was analyzed via flow cytometry. Data from five independent experiments are shown as Points. Statistical analysis was performed using mixed-effects analysis with Tukey′s correction for multiple comparisons. Asterisks indicate significant differences as follows: ns: 0.12, * *p* < 0.033, ** *p* < 0.002, *** *p* < 0.001.

Thus far, our analyses have not revealed a substantial influence of glioblastoma treatment on macrophage activation, immune checkpoint expression, or phagocytosis. Since most of the tumor cells were taken up by the macrophages in less than 2 h, these can be considered short-term effects. To evaluate how tumor treatment might affect the surrounding TME, especially its TAMs, long-term, we performed conditioned medium assays. Therefore, we treated GL261-luc2 glioblastoma cells with different conditions over 5 consecutive days, as described above, with an additional 24 h incubation time. The supernatant was then collected and added to polarized M1- and M2-like macrophages. After another 24 h, the macrophages were collected, and differentiation, activation, and immune checkpoint marker expression were evaluated using flow cytometry. MHC II expression on M1-like cells was significantly increased when the conditioned medium from treated glioblastoma cells was added, with the most prominent effect occurring after chemoradiation (Figure 3A). As expected, the mannose receptor CD206, a marker that identifies M2-like cells, was expressed at significantly higher levels on polarized M2-like cells than on M1-like cells. Interestingly, incubation with conditioned medium from treated tumor cells significantly reduced CD206 surface expression, shifting it to the level observed in M1-like cells (Figure 3B). Prominent alterations were observed in the costimulatory molecules CD80 and CD86. CD80 expression on M1-like macrophages was induced by the tumor cell medium itself, regardless of treatment conditions. For M2-like cells, only a tendency toward increased expression due to treatment conditions was detected (Figure 3C). Induction of CD86 on M1-like cells was even stronger and could be enhanced by RT or combination treatment. No alterations were measured on M2-like cells (Figure 3D).

Next, we analyzed the impact of the microenvironment on the surface expression of immune checkpoint molecules in tumor cells treated with the agent. Neither the mock tumor nor the treated tumor cell medium influenced TIM-3 and OX40L expression (Figure 3E,I). PD-1 receptor expression was more highly expressed on M1-like than on M2-like cells, though no modifications were found depending on the treatment condition of tumor cells (Figure 3G). In contrast, PD-L1 ligand expression was significantly higher on M1-polarized macrophages and increased further when tumor cells underwent treatment, particularly irradiation (Figure 3F). M2-like cells expressed PD-L1 at lower levels than M1-like cells and showed only slight increases with tumor treatment. Furthermore, expression of CD275 is strongly induced on M1-like macrophages by the conditioned tumor cell medium (Figure 3H). Interestingly, irradiation of tumor cells (RT and RCT) significantly reduces this; albeit, the expression levels remain high. By contrast, the expression of CD275 on M2-like cells was not affected by the conditioned medium.

## 3. Discussion

Under physiological conditions, microglia are the primary immune cells responsible for surveillance within the central nervous system, including the brain. In malignancies, up to half of the tumor tissue consists of microglia and macrophages [40]. Macrophages are a heterogeneous cell type with two main subtypes: M1 and M2. These two subtypes exhibit a range of activation patterns. M1 macrophages are considered pro-inflammatory and function to remove bacteria and injured cells. In the context of tumors, they are deemed anti-tumorigenic. In contrast, M2 cells promote tissue repair and are often described as pro-tumorigenic due to their role in tumor proliferation [30,41].

Within gliomas, TAMs can acquire both phenotypes, even though their effector function is mainly suppressive [42], which is one reason why they are often regarded as M2. This results from the immunosuppressive environment created by the tumor, which modifies the attracted macrophages to support tumor growth and keep immunosuppression [43,44]. Targeting chemotaxis pathways to attenuate TAM infiltration into glioblastomas is under current investigation [45]. A 2014 study investigated the correlation between the macrophage subtype and glioma grade, finding that lower expression of the M1-type and higher expression of M2-type macrophages were associated with high-grade glioma [46]. These and other studies demonstrate the presence and correlation of both TAM subtypes (and perhaps others) within the glioma tissue and grade. This information provides insight into the prevailing conditions of the suppressive TME that modulates infiltrating TAMs, and vice versa.

Investigations to date have focused on the infiltration and distribution of TAMs in tumor tissue and strategies for targeting these cells. However, one must consider that tumor cell treatment (a combination of CT (TMZ) with fractionated RT) not only impacts the phenotype of the tumor cells but also modifies infiltrating immune cells. One explanation might be that even seemingly minor changes in the treatment regimen have a significant impact on the immunogenic phenotype of the tumor. With this in mind, we analyzed the effect of a patient-oriented treatment scheme to establish more clinically relevant results. First, we detected no significant alteration in the activation or differentiation of pre-polarized macrophages after contact with tumor cells, regardless of the treatment (Figure 1). This was true for both M1- and M2-like cells. Second, blocking PD-1 had no impact. Anti-PD-1 treatment also did not influence the phagocytosis of treated tumor cells (Figure 2). Other studies have revealed the role of PD-1 in myeloid cells. For example, Gordon et al. found a negative correlation between PD-1 expression on TAMs and phagocytic potency in a CT26 colon carcinoma model [12]. In that study, blocking the PD-1/PD-L1 axis increased the macrophage phagocytosis, reduced tumor growth, and increased survival. However, this could not be observed in our in vitro glioblastoma cell studies. This suggests a subordinate role of the PD-1/PD-L1 interaction in glioblastoma, particularly concerning the function of TAMs. It is most likely that one or more other immunosuppressive factors cover or prevent the activation of PD-1 signaling in glioblastoma TAMs. Additionally, the phagocytosis rate of M1 is supposed to be higher than that of M2 and is thought to promote anti-tumor immunity [47,48]. Although the phagocytic range is relatively broad, we observed a higher phagocytosis rate in M2 cells compared to M1-like cells (Figure 2A,B).

The phagocytic activity of tumor-associated macrophages (TAMs) is shaped by a complex interplay of intrinsic, extrinsic, and tumor microenvironment (TME) factors. Intrinsically, the polarization state of macrophages—whether M1-like or M2-like—is a significant determinant of phagocytic function [49,50]. M1 macrophages exhibit high levels of Fcγ receptors and produce reactive oxygen/nitrogen species (ROS/NOS) and pro-inflammatory cytokines, all of which support efficient phagocytosis, particularly of antibody-opsonized targets [51]. However, in the current setting, opsonization is excluded due to the protocol used in the in vitro model, making receptor expression alone insufficient to predict phagocytic efficacy. The dynamic plasticity of TAMs further complicates interpretation, as subtle deviations in polarization protocols—including cytokine choice, concentration, and exposure time—can result in functional differences even among populations labeled by surface markers as M1 or M2. Furthermore, key extrinsic factors that modulate phagocytosis include “eat me” and “don’t eat me” signals on cancer cells, whereby the balance between these molecules in part determines the outcome of phagocytosis [10]. The balance between these signals seems to differ between M1 and M2 macrophages, but due to the complex plasticity of these cells, no fixed pattern can safely be assigned to the macrophage subtypes. An example of a “don’t eat me” signal is CD47, which is frequently overexpressed by tumor cells. It interacts with SIRPα on macrophages to inhibit phagocytosis; this inhibitory signal is more faithfully respected by M2-like TAMs, contributing to immune evasion [48]. Notably, targeting the CD47-SIRPα interaction enhances phagocytosis [51], a finding also demonstrated in mouse and human M1 and M2 macrophages within a glioblastoma model [48]. But this is only one of many signals. Further research is needed to evaluate the distribution and impact of phagocytic signals in glioblastoma in dependence on treatment, to explain the differences in phagocytic capacity between M1 and M2 macrophages observed in our study. Within the tumor microenvironment, factors such as hypoxia and immunosuppressive cytokines (e.g., IL-10, TGF-β) skew TAMs toward an M2 phenotype and suppress phagocytic function. In contrast, exposure to dying tumor cells or immunogenic stimuli (e.g., DAMPs like HMGB1 and calreticulin) can transiently enhance phagocytosis. In SLE, M2 macrophages are skewed toward M1 macrophages in the presence of HMGB1 and exhibit reduced phagocytosis [52], whereas M2 macrophages were found to be more efficient in clearing apoptotic cells due to phagosome acidification [53].

Furthermore, the type of tumor cell treatment influenced the uptake by M1-like, but not M2-like, macrophages (Figure 2). Temozolomide (CT) treatment enhanced the phagocytosis rate, whereas the combination with RT significantly reduced it. These results suggest a treatment-dependent impact on macrophage phagocytosis, which carries critical clinical implications. First, a reduction in phagocytosis after treatment indicates an unintended immunosuppressive shift within phagocytes and/or the micromilieu, which could undermine the approach of combining immunotherapy with other therapies. Second, the suppression of phagocytosis, in combination with immunosuppression, can promote tumor cell survival and growth. This observation warrants further investigation for its biological relevance in vivo.

From a therapeutic perspective, strategies to reprogram TAMs toward an M1-like state—using checkpoint inhibitors, CSF-1R antagonists, or PI3Kγ inhibitors—aim to restore anti-tumor functions. However, checkpoint blockade alone has shown limited efficacy in this context. Alternatively, directly targeting phagocytosis-inhibitory pathways, including the CD47–SIRPα axis, or activating innate sensing pathways through TLR or STING agonists, offers promising avenues for enhancing macrophage-mediated tumor clearance.

Most interestingly, we discovered that the activation, differentiation, and immunogenicity status of the M1 and M2 macrophage is impacted by the conditioned tumor medium (Figure 3). The most significant influence was observed for M1-like macrophages, for which the tumor cell medium alone strongly induced the expression of the costimulatory molecules CD80 and CD86, as well as MHC II. This effect was further enhanced by treatment; RT and RCT had the most tremendous impact. The changes in immune checkpoint expressions of PD-L1 and CD275 were similar. Overall, these alterations indicate tumor cell and treatment-dependent activation of M1-like macrophages, which could be beneficial for glioblastoma therapy. However, the prominent induction of ICOS-L could be controversial. Upregulation of ICOSLG (in mesenchymal glioma stem cells) in glioblastoma tissue was associated with poor prognosis in patients [54]. In this study, the expression of ICOS-L mediated the expansion of Tregs and IL-10 production, thereby fostering an immunosuppressive environment.

In contrast, the regulatory impact of tumor cells, either alone or depending on their treatment, on M2-like macrophage activation, which most likely mimics TAMs, was relatively small, with a significant induction of CD80. Interestingly, we detected a substantial downregulation of CD206 in polarized M2-like macrophages after RT alone or the combination of RCT. This could hint at a shift from M2-like to M1-like macrophages.

Taken together, the association of M1-like and M2-like macrophages with pro- and anti-tumorigenic responses to glioblastoma is highly context-dependent. Its polarization state is not fixed and is influenced by its environment and vice versa. Further increasing this complexity is the fact that treatment, especially chemoradiation, impacts macrophage polarization and phagocytosis. This has to be investigated further to evaluate real (TAM) targets for successfully combining standard and novel anti-glioblastoma therapies.

## 4. Materials and Methods

### 4.1. Cell Line Culture and Treatment of Glioblastoma Cells

The murine glioblastoma cell line GL261-luc2 (Caliper Life Sciences, Hopkinton, MA, USA) was used as the model system in all experiments. The cell line was cultured in a humidified chamber at 37 °C and 5% CO_2_ in Dulbecco′s Modified Eagle Medium (DMEM, Gibco™, Waltham, MA, USA) supplemented with 10% heat-inactivated fetal calf serum (FCS, Sigma-Aldrich, St. Louis, MO, USA) and 0.5% geneticin (Gibco^TM^, Waltham, MA, USA). For long-term culture, cells were harvested at approximately 80–90% confluence and passaged at a 1:10 ratio in fresh medium, then reseeded into culture flasks. To analyze treatment-related effects, GL261-luc2 cells were seeded in T75 cell culture flasks and incubated overnight. The cells were then treated for 5 consecutive days with either chemotherapy (temozolomide, TMZ) (Sigma-Aldrich, St. Louis, MO, USA), fractionated radiotherapy (RT, 5×2Gy), chemoradiotherapy (combined RCT, chemoradiation), or left untreated as a negative control, depending on their condition. Irradiation was carried out using an X-ray generator (GE Inspection Technologies, Hürth, Germany). Cells were harvested 24 h after the last treatment and used for further analysis.

### 4.2. Animals

C57BL/6 mice were used for the isolation and differentiation of macrophages. The mice were purchased from JANVIER LABS at an age of 6 to 8 weeks and maintained under sterile conditions at the Preclinical Laboratory Animal Facility (PETZ) of the University of Erlangen-Nuremberg. Only male mice were used in all experiments. The local ethics committee approved all experiments.

### 4.3. Generation of M1-like and M2-like Macrophages

All macrophages were bone marrow-derived monocytic progenitors. Briefly, femora and tibia of C57BL/6 mice were prepared, joints were cut, and bone marrow was washed from the bones with macrophage medium (MC medium; DMEM (Gibco™, Waltham, MA, USA)supplemented with 10% FCS (Sigma-Aldrich, St. Louis, MO, USA), 5% horse serum (X), 1% penicillin/streptomycin and 50µM β-mercaptoethanol (both Gibco^TM^, Waltham, MA, USA) via a 27G syringe. A single-cell suspension was prepared by gently pipetting the cell aggregates to disperse them. Bone marrow cells were seeded into a Petri dish and incubated overnight in a humidified chamber at 37 °C and 5%CO_2_. Then, cells were collected, and cell numbers were determined and seeded at a density of 1.5–2 × 10^6^ cells/2 mL MC medium per well. For differentiation of monocytes into M1-like macrophages, cells were stimulated with 4 ng/mL rmGM-CSF (recombinant murine Granulocyte-Macrophage Colony Stimulating Factor) (Miltenyi Biotec, Bergisch Gladbach, Germany), for differentiation into M2 macrophages, 5 ng/mL rmM-CSF (recombinant murine Macrophage Colony Stimulating Factor) (Miltenyi Biotec, Bergisch Gladbach, Germany). Three days later, 0.5 mL of MC medium supplemented with fresh cytokines was added. On day 7, the supernatant from the pre-stimulated macrophages was discarded. Fresh MC medium, including the respective cytokine cocktail, was added: 4 ng/mL rmGM-CSF, one µg/mL LPS (lipopolysaccharide), and 20 ng/mL rmIFN-γ (ImmunoTools GmbH, Friesoythe, Germany) were used to polarize M1-like macrophages; 5 ng/mL M-CSF (Miltenyi Biotec, Bergisch Gladbach, Germany) and 20 ng/mL rmIL-4 (recombinant murine interleukin 4) (ImmunoTools GmbH, Friesoythe, Germany) were used to polarize M2-like macrophages.

### 4.4. Conditioned Medium Assay of M1-like and M2-like Macrophages

One day after polarization, the conditioned medium assay of M1-like and M2-like macrophages was prepared. For this, the cell culture supernatant was collected from the differentially treated GL261-luc2 cells, and 1 mL of conditioned medium was added to the corresponding assay wells of either M1- or M2-like macrophages. The conditioned medium assay was incubated in a humidified chamber at 37 °C and 5% CO_2_ for 24 to 48 h. Polarized macrophages were then washed with ice-cold phosphate-buffered saline (PBS), detached by accutase treatment (both Gibco^TM^, Waltham, MA, USA) (first at 37 °C, then at 4 °C), harvested, and transferred to a canonical tube for flow cytometric analysis.

### 4.5. Phagocytosis Assay

For the phagocytosis assay of glioblastoma cells by M1- and M2-like macrophages, the treated tumor cells were first harvested by trypsinization, collected, and then centrifuged. The cell number was determined, and the cell concentration was adjusted to 1 × 10^6^ cells/mL in TC medium. Subsequently, 3.5 µL of labeling solution (Cell Brite Green, Biotium, Fremont, CA, USA) was added per mL of medium. The cell suspension was then briefly vortexed and incubated at 37 °C for 30 min. Stained tumor cells were washed three times by centrifugation and resuspended in TC medium, and finally resuspended in MC medium at a density of 2 × 10^6^ tumor cells/mL. In a second step, the supernatant was removed from the polarized macrophages, and the cells were washed with ice-cold PBS. They were then detached using Accutase treatment at 4 °C. The number of cells was determined and adjusted to 1 × 10^6^ cells/mL. Next, the phagocytosis assay was set up by adding 1 mL of the macrophage cell suspension to the corresponding treated tumor cell suspension. The assay was incubated for 1 h in a humidified chamber at 37 °C and 5% CO_2_. To stop cellular activity, the assay plates were then placed on ice, and cells were harvested by transferring both the supernatant and the cells into corresponding tubes. To collect cells already attached to the plates, they were washed with PBS, gently removed with a cell scraper, and combined with the cells and supernatant from the appropriate wells. Cells were analyzed by flow cytometric analysis.

### 4.6. Flow Cytometric Analyses

Cells harvested from conditioned medium and phagocytosis assays were washed with cold FACS buffer (PBS containing 10% FCS and 2%NaN3) and centrifuged. The cell pellet was resuspended in Fc blocking buffer (FACS buffer with anti-CD16/32 antibody), incubated at 4 °C for 30 min, and centrifuged. Cells were then stained with different multicolor staining panels. For M1/M2 differentiation and phagocytosis: anti-CD11b, anti-CD206, and ZOMBIE NIR (viability dye) (BioLegend, Inc., San Diego, CA, USA); anti-F4/80, anti-MHCII and anti-CD274 (ThermoFisher Scientific Inc., Waltham, MA, USA); anti-CD80 and anti-CD11c (BD Biosciences, Heidelberg, Germany); anti-CD86 (Miltenyi Biotec, Bergisch Gladbach, Germany). For M1/M2 activation: anti-Ox40L, anti-TIM-3 and anti-PD-1 (ThermoFisher Scientific Inc., Waltham, MA, USA); anti-CD275 and ZOMBIE NIR (BioLegend, San Diego, CA, USA); anti-CD11c (BD Biosciences, Heidelberg, Germany). Flow cytometry was performed using a CytoFLEX and a DxFLEX cytometer (Beckman Coulter Life Sciences, Brea, CA, USA). Data analyses were performed using Kaluza (version 2.1). An exemplary gating strategy for phagocytosis analysis is presented in Supplementary Figure S1A–F. A representative analysis for evaluating surface marker expression is presented in Supplementary Figure S2A–L. The ∆MFI (MFI of target-of-interest minus background fluorescence) was calculated.

### 4.7. Statistical Analysis

Statistical analyses were performed using GraphPad Prism 8. Data were tested for statistical significance using a two-way ANOVA or mixed effects model with Tukey or Bonferroni correction for multiple comparisons. A fitting mixed-effects analysis was used when values were missing. Here, the specified group (M1 or M2 subtype of macrophages) and treatment condition (preM, diffM, mock, CT, RT, RCT) are fixed effects. An interaction term between the group and treatment was also included as a fixed effect. The independent experimental repetitions were modeled as a random effect to account for variability across repeated measures (these were organized as subcolumns in the dataset table). *p* values were set as follows: 0.12 (ns), 0.033 (*), 0.002 (**), <0.001 (***).

## Data Availability

All data sets and statistical analyses generated and used in this study are available upon request.

## Supplementary Materials

The following supporting information can be downloaded at: https://www.mdpi.com/article/10.3390/ijms26146574/s1.

## Abbreviations

The following abbreviations are used in this manuscript:

BBB	blood–brain barrier
C57BL/6	wildtype mouse strain
CD	cluster of differentiation
CNS	central nervous system
CT	chemotherapy
CTLA4	Cytotoxic T Lymphocyte Antigen 4
DC	dendritic cell
ICOS-L	Inducible costimulator-ligand
IFN-γ	interferon-gamma
LPS	lipopolysaccharide
MDSC	myeloid-derived suppressor cell
MFI	mean fluorescence intensity
MHC II	Major Histocompatibility Complex II
OS	overall survival
OX40L	CD252
PD-1	Programmed Cell Death Protein 1
PD-L1	Programmed Cell Death Protein Ligand 1
PFS	progression-free survival
RCT	combination of RT and CT/chemoradiation
RT	radiotherapy
TAM	tumor-associated macrophage
TIM-3	T cell immunoglobulin and mucin-domain containing-3
TME	tumor microenvironment
TMZ	temozolomide
Treg	regulatory T cell
