# Supplementary material for: Chemoradiation-Altered Micromilieu of Glioblastoma Cells Particularly Impacts M1-like Macrophage Activation
Source: Int J Mol Sci. 2025 Jul 8;26(14):6574. doi: 10.3390/ijms26146574 (PMC12294741; doi:10.3390/ijms26146574)
Supplement: Supplementary file 1 [file ijms-26-06574-s001.zip › ijms-3707190-supplementary.pdf]

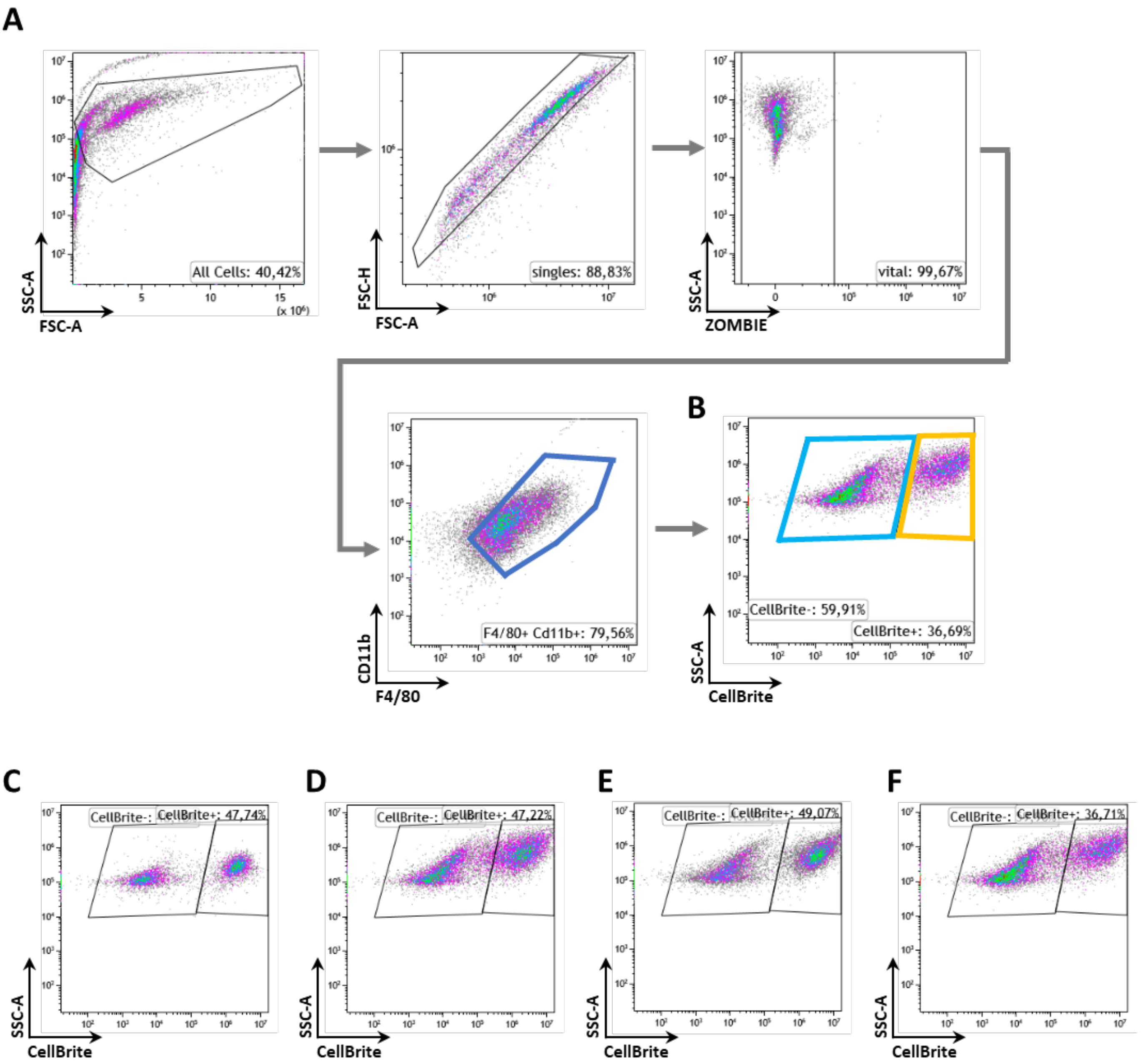

Supplementary Figure S1: Representative gating strategy for GL261-luc2 phagocytosis by M1-like macrophages. (A) Pre-gating excluded debris from whole cells and chose singlets based on FSC-A vs. FSC-H , followed by exclusion of dead cells (ZOMBIE-NIR$^+$). Next, cells gated for F4/80 and CD11b expression were considered to be macrophages. (B) The differentiation between CellBrite$^-$ and CellBrite$^+$ cells distinguished between macrophages alone (CellBrite$^-$) and macrophages that phagocytosed tumor cells (CellBrite$^+$). Exemplary gating of phagocytosed (C) mock, (D) CT-, (E) RT -and (F) RCT– pre-treated GL261-luc2 cells.

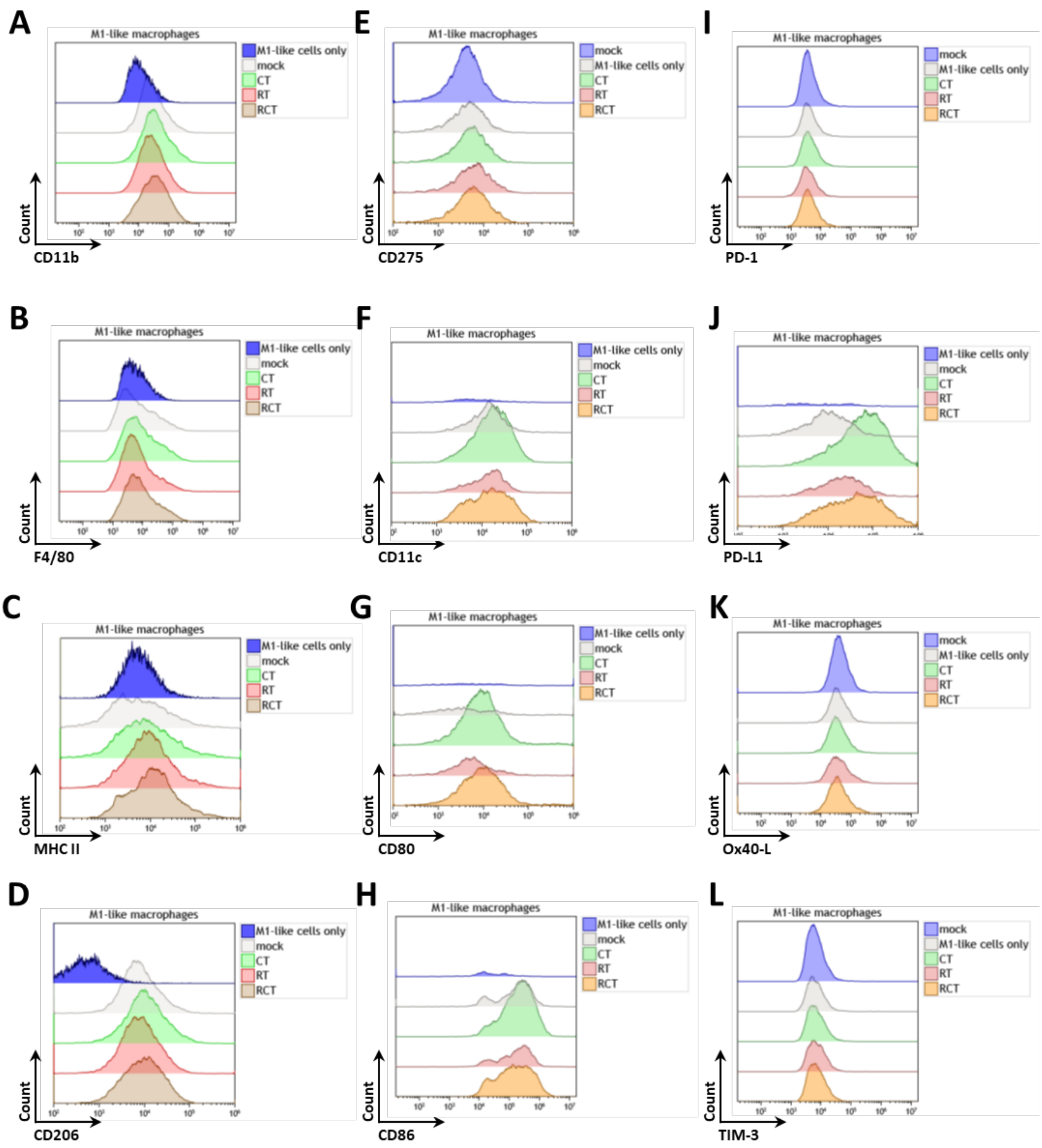

Supplementary Figure S2: Representative histogram overlay of M1-like macrophage expression analyses after tumor cell contact. Pre-gating excluded debris from whole cells and selected singlets based on FSC-A vs. FSC-H , then excluded dead cells (ZOMBIE-NIR$^+$). Vital cells (ZOMBIE-NIR$^-$) that were gated positive for F4/80 and CD11b expression were considered macrophages. The surface expression of the following markers was analyzed: (A) CD11b, (B) F4/80, (C) MHC II, (D) CD206, (E) CD275, (F) CD11c, (G) CD80, (H) CD86, (I) PD-1, (J) PD-L1, (K) OX40-L, and (L) TIM-3. The histograms are presented in colors dependent on the condition and treatment of macropahges; blue histograms: M1-like cells only; grey, green, red, and orange: co-Culture condition of M1-macrophages with mock-, CT-, RT– and RCT-treated glioblastoma cells.
